# Third international conference “Bioinformatics: from Algorithms to Applications” (BiATA 2019): Saint Petersburg, Russia. 20-22 June 2019

**DOI:** 10.1186/s12859-019-3122-9

**Published:** 2019-11-08

## Anton Korobeynikov (biata@spbu.ru), Alla Lapidus (biata@spbu.ru)

### Center for Algorithmic Biotechnologies, Saint Petersburg State University, Saint Petersburg, Russia, 199034

The III International Conference "Bioinformatics: from Algorithms to Applications" (BiATA2019) has established itself as one of the few conferences in the field of bioinformatics that brings together both the programmers creating tools for modern studies in multiple areas of life sciences and the researchers conducting those experiments interested in finding reliable and easy to use tools for data analysis.

BiATA gives the international community a platform to present the latest achievements in bioinformatics and provides an excellent opportunity for researchers to discuss their pressing needs directly with software developers, while demonstrating the results they have already managed to achieve. This type of interaction is completely unique, because algorithm authors and users habitually tend to attend completely different conferences, which prevents this kind of information exchange.

The conference aims to popularize bioinformatics and promotes active application of bioinformatics in agricultural and biomedical fields of research; identify new trends in the fields of bioinformatics, computational genomics and transcriptomics, as well as in sequencing of biologically active molecules and the application of mathematical methods and algorithms in the life sciences.

Topics covered within the framework of the conference include but are not limited to:

1. Algorithms for the assembly of metagenomic data
2. Big data metagenomics
3. New algorithms for assembling and analyzing long reads obtained via new sequencing technologies
4. Computer biology and agriculture: analysis of soil and air microbiota
5. Human microbiota: nutrition and health
6. Bioinformatics of virome

The event also pays a great deal of attention to the most important task of all genomic research - restoring the primary sequence of genomic DNA from short fragments obtained as a result of using modern DNA sequencing technologies. Despite the fact that the restoration of the primary structure of DNA is not in itself the ultimate goal of research, all subsequent analyses depend on its quality. The quality of genome assembly becomes even more important when dealing with sequencing data generated from the combined genome of natural communities of microorganisms (microbiota) that inhabit a variety of different natural environments (soil, water, air, plants, etc.). Metagenomics - analytical methods and approaches that allow studying total genomes (microbiomes) - deals with large volumes of very complex data and requires specialized methods for solving scientific problems in such important areas as agriculture, medicine, etc.

The timeliness of the subject matter and the high level of quality of the conference can be evidenced by the level of speakers who took part in BiATA (http://biata2019.spbu.ru/).

The conference brought together more than 100 participants from Russia, Belgium, Canada, China, Great Britain, France, Israel, Italy, Japan, Latvia, Lebanon, Spain, Singapore and the USA.

## O1 Probabilistic model of CDR3 junctions formation in human Ig heavy chain genes and its application

### Evgeny A. Bakin^1^, Elena A. Pazhenkova^2^, Oksana V. Stanevich^3^

#### ^1^Bioinformatics Institute, Saint Petersburg, Russia, 197342; ^2^Saint Petersburg University, Saint Petersburg, Russia, 199034; ^3^Smorodintsev Research Institute of Influenza, Saint Petersburg, Russia, 197376

##### **Correspondence:** Evgeny A. Bakin (evgeny.bakin@bioinf.me)

Immunoglobulins (Igs) play a crucial role in the adaptive immune system. Igs are composed of polypeptide subunits: light and heavy chains. The latter contains a variable domain that is important for an antigen binding. The coding sequences for IG heavy chain are produced through a complex process, including VDJ recombination and somatic hypermutation (SHM). The latter masks initial segments, which complicates a precise sequence analysis of Ig genes in B-cells. Thus, in this research we focus on such a robust parameter of Ig genes sequence as the length of a V-D/D-J junction, which strongly influences antibodies affinity. As is known, these junctions may be subject to an abnormal recombination, sometimes leading to an autoreactivity and a subsequent lymphomagenesis (e.g. due to VH-replacement). Initially, a junction consists of palindromic (p)-nucleotides (produced by a protein complex of Ku70/Ku80 and Artemis) and non-templated (n)-nucleotides (added by a TdT protein), which further undergoes an impact of exo- and endonucleases.

For all the three stages of V-D junction maturation, we propose simple, yet tractable probabilistic models resulting in a general model describing a distribution of a junction lengths in normal immunoglobulins. The parameters for the developed model may be fitted by means of datasets obtained from healthy individuals, which are available in open databases such as GenBank and ENA. For this purpose, we have developed a pipeline containing the following steps:

1. Ig genes repertoire assembly (pRESTO);

2. clonal families detection and data decorrelation (Partis);

3. sequences demarcation and V-D/D-J junctions extraction (IMGT HighV-QUEST);

4. fitting model parameters via maximum likelihood estimation (custom Python scripts).

An evaluation of the model showed its consistency with the processed samples. The trained model was further applied to datasets describing Ig genes sequences with abnormalities in VDJ process. For this data a statistically significant divergence with the model was detected. At the same time, no divergence was detected for diseases not related to onco-hematology. This experiment has shown that a V-D/D-J junction length distribution in Ig repertoire may be used as an indicator of the presence of pathological clones in a B-cell population. The possibility of the model application as an early predictor of various diseases presents a significant interest for further research.

## O2 Bacteriophage recombination site helps to reveal genes potentially acquired through horizontal gene transfer

### Maria A Daugavet^1^, Sergey V Shabelnikov^1^, Leonid S Adonin^1^, Olga I Podgornaya^1,2^

#### ^1^Institute of Cytology, St. Petersburg, Russia, 194064; ^2^School of Biomedicine, Far Eastern Federal University, Vladivostok, Russia, 690090

##### **Correspondence:** Maria A Daugavet (ka6tanka@yandex.ru)

**Background** The cellulose-synthase gene of ascidians was gained from prokaryote donor and this is the most reliable example of the horizontal gene transfer (HGT). In our previous study a new protein, rusticalin, of ascidian *Styela rustica* was described. Its C‑terminal domain coding region was also shown to be inherited from prokaryote ancestor by means of HGT. Both for rusticalin C‑terminal domain and for cellulose-synthase catalytic domain it was shown that there coding regions neighbored with bacteriophage recombination site AttP. Thus we suggested a possible mechanism of HGT by means of bacteriophage insertion. Most of the cases of HGT are described based on sequence similarity alone, but in case of rusticalin we also demonstrated strong evidence of the mechanism of transfer by identifying the recombination site.

**Results** It is possible that bacteriophage recombination site can help finding yet other new cases of HGT in eukaryotic genomes. Unfortunately the length of bacteriophage recombination site AttP is 43 nucleotides which is too short to find it reliably in big databases. Still we know that in rusticalin related gene AttP-like site is situated inside the cysteine-rich repeats coding region. Based on that we performed a remote similarity search HMMER using amino acid sequence of cysteine-rich repeats. Cysteine-rich repeats appeared to be part of larger proteins. Therefore conserved domains associated with cysteine-rich repeats were classified.

In spite of the fact that cysteine-rich repeats are found almost exclusively in eukaryotic proteins, they are usually associated with domains typical for prokaryotes or bacteriophages (in 98 proteins out of 124). Among them in 20% (26 proteins) cysteine-rich repeats are associated with phage-lysozyme (PF00959), 14% (17 proteins) with amidase_2 (PF01510). In general nine different domains associated with cysteine-rich repeats can be classified as bacterial cell-wall hydrolyzing enzymes. It is worth to mention that phage-lysozyme domain is found together with cysteine-rich repeats in proteins of different species and even of different taxa as Fungi and Metazoa.

**Conclusions** Based on that observations we can conclude that cysteine-rich repeat in Eukaryotic proteins is usually accompanied by typical prokaryotic domains. The explanation of that might be the presence of bacteriophage recombination site inside cysteine-rich repeat coding sequence, which can facilitate HGT. The 98 genes potentially acquired through HGT from prokaryotes is found as the result.

**Funding:** The work was supported by program “Molecular and cell biology” of the Russian Academy of Sciences and RSF (19-74-20102).

## O3 SPAligner: alignment of long diverged molecular sequences to assembly graphs

### Tatiana Dvorkina^1^, Dmitry Antipov^1^, Anton Korobeynikov^1,2^ and Sergey Nurk^1^

#### ^1^Center for Algorithmic Biotechnology, St. Petersburg State University, St. Petersburg, Russia; ^2^Department of Statistical Modelling, St. Petersburg State University, St. Petersburg, Russia

##### **Correspondence:** Tatiana Dvorkina (tanunia@gmail.com)

Many popular short read assemblers [9,10,11] provide the user not only with a set of contig sequences, but also with *assembly graphs*, encoding the information on the potential adjacencies of the assembled sequences. Naturally arising problem of sequence-to-graph alignment has been a topic of many recent studies [2,3,5,6,7,8,13,14]. Identifying alignments of long error-prone reads (such as Pacbio and ONT reads) to assembly graphs is particularly important and has recently been applied to hybrid genome assembly [1,4], read error correction [12], and haplotype separation [3]. At the same time, the choice of the practical aligners supporting long nucleotide sequences is currently limited to vg [2] and GraphAligner[3], both of which are under active development. Moreover, to the best of our knowledge, no existing graph-based aligner supports alignment of amino acid sequences.

Here we present the SPAligner tool for aligning long diverged molecular (both nucleotide and amino acid) sequences against assembly graphs produced by the popular short-read assemblers. The project stemmed from our previous efforts on the long-read alignment within the hybridSPAdes assembler [1]. Our benchmarks on various Pacbio and Oxford Nanopore datasets show that SPAligner is highly competitive to vg and GraphAligner in aligning long error-prone reads. We also demonstrate SPAligner’s ability to accurately align amino-acid sequences (with up to 80% amino acid identity) onto complex assembly graphs of metagenomic datasets. To further motivate this application we show how SPAligner can be used for identification of biologically important (antibiotic-resistance) genes, which remain under the radar of conventional pipelines due to assembly fragmentation (e.g. genes exhibiting high variability in complex environmental samples). SPAligner is available for download at http://cab.spbu.ru/software/spaligner/

**References**

1. Antipov D, Korobeynikov A, McLean JS, Pevzner PA. hybridSPAdes: an algorithm for hybrid assembly of short and long reads. Bioinformatics. 2015 Nov 20;32(7):1009-15.

2. Garrison E, Sirén J, Novak AM, Hickey G, Eizenga JM, Dawson ET, Jones W, Garg S, Markello C, Lin MF, Paten B. Variation graph toolkit improves read mapping by representing genetic variation in the reference. Nature biotechnology. 2018 Aug 20.

3. Garg S, Rautiainen M, Novak AM, Garrison E, Durbin R, Marschall T. A graph-based approach to diploid genome assembly. Bioinformatics. 2018 Jun 27;34(13):i105-14.

4. Wick RR, Judd LM, Gorrie CL, Holt KE. Unicycler: resolving bacterial genome assemblies from short and long sequencing reads. PLoS computational biology. 2017 Jun 8;13(6):e1005595.

5. Heydari M, Miclotte G, Van de Peer Y, Fostier J. BrownieAligner: accurate alignment of Illumina sequencing data to de Bruijn graphs. BMC bioinformatics. 2018 Dec;19(1):311.

6. Ye Y, Tang H. Utilizing de Bruijn graph of metagenome assembly for metatranscriptome analysis. Bioinformatics. 2015 Aug 29;32(7):1001-8.

7. Limasset A, Flot JF, Peterlongo P. Toward perfect reads: self-correction of short reads via mapping on de Bruijn graphs. arXiv preprint arXiv:1711.03336. 2017 Nov 9.

8. Kavya VN, Tayal K, Srinivasan R, Sivadasan N. Sequence alignment on directed graphs. Journal of Computational Biology. 2019 Jan 1;26(1):53-67.

9. Bankevich A, Nurk S, Antipov D, Gurevich AA, Dvorkin M, Kulikov AS, Lesin VM, Nikolenko SI, Pham S, Prjibelski AD, Pyshkin AV. SPAdes: a new genome assembly algorithm and its applications to single-cell sequencing. Journal of computational biology. 2012 May 1;19(5):455-77.

10. Li D, Liu CM, Luo R, Sadakane K, Lam TW. MEGAHIT: an ultra-fast single-node solution for large and complex metagenomics assembly via succinct de Bruijn graph. Bioinformatics. 2015 Jan 20;31(10):1674-6.

11. Chikhi R, Rizk G. Space-efficient and exact de Bruijn graph representation based on a Bloom filter. Algorithms for Molecular Biology. 2013 Jan;8(1):22.

12. Salmela L, Rivals E. LoRDEC: accurate and efficient long read error correction. Bioinformatics. 2014 Aug 26;30(24):3506-14.

13. Jain C, Zhang H, Gao Y, Aluru S. On the complexity of sequence to graph alignment. InInternational Conference on Research in Computational Molecular Biology 2019 May 5 (pp. 85-100). Springer, Cham.

14. Rautiainen M, Marschall T. Aligning sequences to general graphs in O (V+ mE) time. bioRxiv. 2017 Jan 1:216127.

## O4 HEDGE: GPU-powered protein-protein docking pipeline with accurate energy metrics as a primary scoring function

### Timofei Ermak, Artem Shehovtsov, Pavel Yakovlev

#### BIOCAD, Saint Petersburg, Russia

##### **Correspondence:** Timofei Ermak (timofei.ermak@live.com)

Protein-protein interactions play key roles in living systems: cell signaling, immune system reactions, microelements transport and many other processes are based on protein-protein complexes functions. Thus, protein-protein complexes prediction is very important task especially in terms of drug discovery. For instance, *in silico* optimization stages of antibody-based drug development process requires to solve the problem hundreds times. To perform *in silico* optimization accurately the docking problem must be solved with high accuracy in short time ranges. But it is one of the hardest, both methodologically and computationally, structural bioinformatics problems.

Recently we developed a pipeline called HEDGE, briefly it can described as follows: 1) scanning translational solution space using FFT correlation theorem with energy-like correlation function; 2) clustering of solutions by RMSD as a distance metric; 3) refinement of full complex structures with minimization of potential energy: Polak-Ribière-Polyak conjugate gradient method [1] is used to solve optimization problem, optimization target is OPLS [2] force field with additional GB and SA terms; 4) Finally we rank solutions by change of Gibbs free energy (ΔG), which can be considered as the most accurate metric to rank predicted complexes.

Each step of the pipeline above is well-parallelizable, so, the full power of GPUs (graphics processing units) is utilized, thus, overall computation time decreased significantly. Moreover, different rotations of molecules can be processed independently, therefore, multi-GPU mode is supported to scale linearly and achieve maximal performance on multi-GPU supercomputers.

Accuracy was tested on a subset of CAPRI [3] dataset showing about 50% of correct predictions. Time required for prediction of one complex in rigid mode (without minimization) is about 7 minutes on Tesla V100 GPU. Flexible mode requires much more calculations and takes about 1.5 hours on Tesla V100.

**References**

1. Polak, Elijah, and Gerard Ribiere. "Note sur la convergence de méthodes de directions conjuguées." *Revue française d'informatique et de recherche opérationnelle. Série rouge*3.16 (1969): 35-43.

2. Robertson, Michael J., Julian Tirado-Rives, and William L. Jorgensen. "Improved peptide and protein torsional energetics with the OPLS-AA force field." *Journal of chemical theory and computation* 11.7 (2015): 3499-3509.

3. Janin, Joel. "Welcome to CAPRI: a critical assessment of predicted interactions." *Proteins: Structure, Function, and Bioinformatics* 47.3 (2002): 257-257.

## O5 Million sequences indexing

### Antoine Limasset^1,2^

#### ^1^Department of Computer Science, Research Center in Computer Science, Signal and Automatic Control of Lille (CRIStAL), Lille, France; ^2^National Centre of the Scientific Research (CNRS), Lille, France

Most methodological contributions handling sequencing data now acknowledge the need to scale up to the terrific throughput that we face nowadays. Since BLAST, a plethora of tools have been developed to handle the massive amount of available reference sequences. Recently, new structures have been proposed to link a short sequence such as a transcript or a gene to sequencing datasets or reference genomes. The challenge of such structures is to be able to index hundreds of thousands of datasets with a reasonable amount of memory while being able to perform fast queries. A prosperous state of the art of efficient tools quickly emerged, SBT SSBT, HowDeSBT, BIGSI, … based on different combinations of bloom filters in order to link a k-mer to its associated datasets. Those approaches are incredibly efficient: BIGSI was able to index half a million bacterial genomes with 1.5TB. However, not able to scale to all known genomes or all transcriptomes collections. We aim to propose a new data structure that could use an order of magnitude less memory than BIGSI while being able to perform similar queries in terms of accuracy and throughput. Instead of indexing all k-mers of a dataset, we choose to rely on local sensitive hashing methods to index a small subset of the input k-mers. This choice allows the scaling of the methods with a satisfying accuracy on medium-sized queries (1kb or larger). Furthermore, we rely on a matrix-like structure similar to BIGSI, that offer fundamental properties for such an index:

● Constant time insertion of a new reference sequence by adding a new row to the matrix

● Queries rely on reading columns that can be compressed column for lighter structure and faster queries

● Easy to balance structure where memory/accuracy trade-off can be precisely chosen

In this presentation, we show the design of such a structure using the Min-Hash scheme. We present preliminary results on a hundred thousand bacterial genomes on a proof of concept implementation. We compare our performances to BIGSI and Mashscreen, showing that the proposed structure can achieve a comparable accuracy with a better scaling in memory or throughput. We finally discuss the incoming improvements and what can potential pitfalls from this scheme, and its potential applications in mega-scale sequences indexing, clustering, or genome assembly. The open-source implementation is under development and available on Github at https://github.com/Malfoy/Miekki

## O6 Revealing mechanisms of *Bacillus thuringiensis* host specificity via molecular modelling of the Cry toxin-receptor interactions

### Yury V. Malovichko^1,2^, Anton E. Shikov^1,2^, Rostislav K. Skitchenko^3^, Anton A. Nizhnikov^1,2^, Kirill S. Antonets^1,2^

#### ^1^Laboratory for Proteomics of Supra-Organismal Systems, All-Russia Research Institute for Agricultural Microbiology (ARRIAM), St. Petersburg, Russia; ^2^Faculty of Biology, St. Petersburg State University, St. Petersburg, Russia; ^3^Faculty of Applied Optics, ITMO University, St. Petersburg, Russia

##### **Correspondence:** Yury V. Malovichko (yu.malovichko@arriam.ru) and Kirill S. Antonets (k.antonets@arriam.ru)

Proteins possessing cytotoxic properties and commonly referred to simply as toxins comprise a vast group of bacterial virulence factors. For instance, *Bacillus thuringiensis*, a spore-forming bacterial pathogen of insects and several other invertebrate taxa, produces at least four major classes of proteinaceous toxins. Of these, crystalline pore-forming toxins produced at sporulation stage and known as Cry toxins, pose a particular interest because of the wide range of host species they affect. Cry toxins consist of three domains flanked with unstructured terminal sequences, with the N-terminal domain participating in pore formation and the other two involved in binding to a respective host’s receptor. To date, more than 700 Cry toxins affecting species of four Insecta orders as well as those of Nematoda phylum have been discovered, and several insect membrane proteins, have been proposed to serve as Cry receptors; however, little is known about the molecular mechanisms underlying both mode of action and specificity of these toxins. In this work, we analyze coevolutionary substitution patterns in toxins and alanyl aminopeptidase receptors to reveal molecular mechanisms of the specificity of toxin-receptor interactions. To enlarge the toxin dataset, we developed a novel HMM-based tool for searching Cry toxins and annotating their domain structure, which outperforms its analogs. Launching this tool with all *Bacillus*-related sequences we discovered 340 new toxins and defined domain layout of all known Cry proteins. Next, amino acid substitutions distinguishing protein sequences in both toxin and receptor epitope subsets were mapped onto minimum distance transition graphs which were then aligned to expose similarities in their topology. Subsequent *in silico* docking of toxins and insect N-alanyl aminopeptidases revealed sites unequivocally involved in toxin-receptor interactions and effects of amino acid substitutions in these sites. Obtained data might be useful for designing novel Cry toxins efficient against particular hosts.

This work was supported by the Russian Science Foundation (Grant No 18-76-00028).

## O7 Indexing De Bruijn graphs with minimizers

### Camille Marchet, Maël Kerbiriou and Antoine Limasset

#### Univ. Lille, CNRS, Inria, Lille, France

##### **Correspondence:** Camille Marchet (camille.marchet@univ-lille.fr)

The need to associate information to words is shared among a plethora of applications and methods in high throughput sequence analysis and became fundamental. However, indexing billions of *k*-mers may lead to scalability issues, as exact associative indexes can be memory expensive. To leverage this issue, recent works take advantage of the properties of the *k*-mer sets [1]. They exploit the overlaps shared among *k*-mers by using a De Bruijn graph as a compact *k*-mer set to provide lightweight structures.

Contribution: we propose a scalable and exact index structure able to associate unique identifiers to indexed *k*-mers and to reject alien *k*-mers. The proposed index combines an extremely compact representation along with high throughput. Moreover, it can be efficiently built from the De Bruijn graph sequences. The index implementation we provide achieved to index the *k*-mers from the human genome with 8GB within 30 minutes, and was able to scale up to the enormous axolotl genome (32 Gbp [2]) with 63GB within 10 hours. Furthermore, while being memory efficient, the index allows above a million queries per second on a single CPU in our experiments, and its throughput can be raised using multiple cores. Finally, we also present the index ability to practically represent metagenomic and transcriptomic sequencing data to highlight its wide applicative range.

Availability: the index is implemented as a header-only library in C++, is open source under AGPL3 license and available at https://github.com/Malfoy/Blight. It is designed as a user-friendly library and comes along with sample code usage.

**References**

1. Almodaresi F, Pandey P, Ferdman M, Johnson R, Patro R. An Efficient, Scalable and Exact Representation of High-Dimensional Color Information Enabled via de Bruijn Graph Search. International Conference on Research in Computational Molecular Biology. 2019; 1-18.

2. Nowoshilow S, Schloissnig S, Fei J-F. The axolotl genome and the evolution of key tissue formation regulators. Nature. 2018, 554(7690): 50.

## O8 Synteny paths for assembly graphs comparison

### Evgeny Polevikov^1^ and Mikhail Kolmogorov^2^

#### ^1^Bioinformatics Institute, St. Petersbrug, Russia; ^2^Department of Computer Science and Engineering, University of California, San Diego, CA, USA

##### **Correspondence:** Mikhail Kolmogorov (mkolmogo@ucsd.edu)

Despite the recent developments of long-read sequencing technologies, it is still difficult to produce complete assemblies of eukaryotic genomes in an automated fashion [1]. Genome assembly software typically output assembled fragments (contigs) along with assembly graphs, that encode all possible layouts of these contigs. Graph representation of the assembled genome can be useful for gene discovery, haplotyping, structural variations analysis and other applications. To facilitate the development of new graph-based approaches, it is important to develop algorithms for comparison and evaluation of assembly graphs produced by different software

In this work, we introduce *synteny paths*: maximal paths of homologous sequence between the compared assembly graphs. We describe Asgan – an algorithm for efficient synteny paths decomposition, and use it to evaluate assembly graphs of various bacterial assemblies produced by different approaches. Similarly to synteny blocks [2] (that are commonly used in comparative genomics studies), synteny paths reveal structural similarities between the compared genomes, but are robust to assembly fragmentation. We define the problem of finding the minimum number of synteny paths between two assembly graphs and prove that the exact solution is NP-hard. Instead, we propose an efficient approximate algorithm for synteny path decomposition via transforming two assembly graphs into a colored breakpoint graph.

First, we use synteny paths to compare between Flye [3] and Canu [4] assemblies of 21 bacterial genomes from the NCTC collection. The final graphs produced by two assemblers from the same genome might not be identical, due to the different approaches to repeat resolution. However, if graphs are free from errors, they should encode the original genome sequence as path. Indeed, in 14 out of 21 NCTC datasets each connected component in assembly graphs was covered by a single synteny path, revealing the putative genome walks.

Secondly, we apply synteny paths to compare between the assemblies of 15 *Drosophila* genomes with extensive structural variations. We show that synteny paths reveal longer homologous segments, comparing to synteny blocks reconstructed using the fragmented contigs. Interestingly, the length distribution of synteny paths was highly correlated with the evolutionary distances between the compared genomes. This allowed to reconstruct the phylogenetic tree of 15 *Drosophila* genomes using pairwise synteny paths similarities as a distance metric.

**References:**

1. Mitchell R Vollger, Philip C Dishuck, Melanie Sorensen, AnneMarie E Welch, Vy Dang, Max L Dougherty, Tina A Graves-Lindsay, Richard K Wilson, Mark JP Chaisson, and Evan E Eichler. Long-read sequence and assembly of segmental duplications. *Nature methods*, 16(1):88, 2019.

2. Pavel Pevzner and Glenn Tesler. Transforming men into mice: the Nadeau-Taylor chromosomal breakage model revisited. In Proceedings of the seventh annual international conference on Research in computational molecular biology, pages 247–256. ACM, 2003.

3. Mikhail Kolmogorov, Jeffrey Yuan, Yu Lin, and Pavel A Pevzner. Assembly of long, error-prone reads using repeat graphs. Nature biotechnology , 37(5):540, 2019.

4. Sergey Koren, Brian P Walenz, Konstantin Berlin, Jason R Miller, Nicholas H Bergman, and Adam M Phillippy. Canu: scalable and accurate long-read assembly via adaptive k-mer weighting and repeat separation. Genome research, 27(5):722–736, 2017.

## O9 Extending rnaSPAdes functionality for hybrid transcriptome assembly

### Andrey Prjibelski^1^, Elena Bushmanova^1^, Daniela Giordano^3^, Alla Mikheenko^1^, Guiseppe Puglia^2^, Dmitry Antipov^1^, Domenico Viatale^2^, Alla Lapidus^1^

#### ^1^ Center for Algorithmic Biotechnology, Institute of Translational Biomedicine, St. Petersburg State University, St. Petersburg, Russia; ^2^ Consiglio Nazionale delle Ricerche, Istituto per i Sistemi Agricoli e Forestali del Mediterraneo, Catania, Italy; ^3^ University of Catania, Catania, Italy

##### **Correspondence:** Andrey Prjibelski (a.przhibelsky@spbu.ru)

*De novo* RNA-Seq assembly is a powerful method for analysing transcriptomes when the reference genome is not available or poorly annotated. However, due to the short length of Illumina reads it is often impossible to reconstruct complete sequences of complex genes and alternative isoforms. Recently emerged possibility to generate long RNA reads, such as PacBio and Oxford Nanopores, may dramatically improve the assembly quality, and thus the consecutive analysis. While reference-based pipelines were already developed and applied to long RNA reads [1, 2], there are not many possibilities for *de novo* assembly of such data. Among available methods, Trinity [3] supports long error-corrected reads as an input, and IDP-denovo [4] performs hybrid transcriptome assembly using long reads and contigs generated from short-read data by any third-party assembler.

In this work we present a novel algorithm that allows to perform high-quality *de novo* transcriptome assemblies by combining accuracy and reliability of short reads with exon structure information from long error-prone reads. The algorithm is designed by incorporating existing hybridSPAdes approach [5] into rnaSPAdes pipeline [6] and adapting it for transcriptomic data. Since in some cases long-read technologies allow to derive full-length (FL) mRNA sequences from raw reads based on terminal adapters, the developed method additionally supports FL reads as an input, which further helps to determine complete isoform sequences.

To evaluate the benefit of using long RNA reads we use several datasets containing both Illumina reads and long reads obtained by Iso-seq or ONT technologies. Using existing quality assessment software, we compare short-read and hybrid assemblies generated by the new version of rnaSPAdes, as well as Trinity and IDP-denovo.

**References**

1. Garalde, Daniel R., et al. "Highly parallel direct RNA sequencing on an array of nanopores." Nature methods 15.3 (2018): 201.

2. Pacific Biosciences. (2014). Intro to the Iso-Seq Method: Full-length transcript sequencing. June 2, 2014. https://www.pacb.com/blog/intro-to-iso-seq-method-full-leng

3. Grabherr, Manfred G., et al. "Full-length transcriptome assembly from RNA-Seq data without a reference genome." Nature biotechnology 29.7 (2011): 644.

4. Fu, Shuhua, et al. "IDP-denovo: de novo transcriptome assembly and isoform annotation by hybrid sequencing." Bioinformatics 34.13 (2018): 2168-2176.

5. Antipov, Dmitry, et al. "hybridSPAdes: an algorithm for hybrid assembly of short and long reads." Bioinformatics 32.7 (2015): 1009-1015.

6. Bushmanova, Elena, et al. "rnaSPAdes: a de novo transcriptome assembler and its application to RNA-Seq data." bioRxiv (2018): 420208.Bushmanova, E. et al, 2018. rnaSPAdes: a de novo transcriptome assembler and its application to RNA-Seq data. bioRxiv, p.048942.

## O10 PathRacer: racing profile HMM paths on assembly graph

### Alexander Shlemov^1^, Anton Korobeynikov^1, 2^

#### ^1^Center for Algorithmic Biotechnology, St. Petersburg State University, St. Petersburg, Russia; ^2^Department of Statistical Modelling, St. Petersburg State University, St. Petersburg, Russia

##### **Correspondence:** Anton Korobeynikov (a.korobeynikov@spbu.ru)

Recent studies resulted in large databases that store profile Hidden Markov Models (pHMMs) representing different genes families including the families of antibiotic resistance genes, CRY gene domains, biosynthetic gene clusters, or allelic variations amongst highly conserved housekeeping. However, the effective use of these databases for the gene search from genome assemblies might be limited as there is the inherit requirement that the sequence of gene of interest should reside within the single contig. Such a condition is often violated for metagenome assemblies preventing the further analysis.

We present SPHMM – a suite of tools aimed for solving various pHMM alignment problems. SPHMM consists of PathRacer-Graph – a novel standalone tool that performs profile HMM to the assembly graph alignment (necessary codon translation is performed along the alignment process for amino acid pHMMs). PathRacer-Graph yields the set of most probable paths traversed by a HMM through the assembly graph, regardless whether the sequence of interested is located on the single contig or scattered across the set of edges, therefore significantly improving the recovery of sequences of interest even from fragmented metagenome assemblies.

Another member of SPHMM family is PathRacer-Seq that produces frameshift-tolerant alignments of amino acid pHMM to nucleotide sequences significantly improving the accuracy of gene recovery out of assemblies obtained from long noisy reads.

## O11 Local sequence alignment using intra-processor parallelism

### Dmitry Orekhov^1,2^, Alexander Tiskin^3^

#### ^1^St Petersburg University, Russia; ^2^Bioinformatics Institute, St Petersburg, Russia; ^3^University of Warwick, Coventry, United Kingdom

Local alignment of DNA sequences is a fundamental problem of bioinformatics. Standard solutions include fast heuristics, as well as the more time-consuming exact methods. An efficient exact local alignment technique, based on a “sliding window” approach, was previously developed at Warwick [1], resulting in biologically significant results [2-4]. The efficiency of that implementation was achieved, in particular, by low-level intra-processor parallelism.

In recent years, microprocessor architecture has been developing rapidly, culminating with Intel’s AVX-512 [5], an instruction set taking intra-processor parallelism to a new level of efficiency and sophistication, while also being surprisingly well-suited for speeding up the “braid combing” sequence alignment technique developed by the second author [6-7]. We present a prototype software tool [8] that is, to our knowledge, the first sequence alignment software taking advantage of AVX-512 parallelism. Our approach allows one to produce sliding window alignments between a short fragment (“pattern”) and a long sequence (“text”), using braid combing and intra-processor parallelism.

In the simplest case of unweighted alignment, the braid combing algorithm can be described as growing an object called a braid, embedded in the grid defined by the input sequences (Figure 1). The combing logic is as follows: we iterate over cells of the grid left-to-right and top-to-bottom, extending the braid to the current cell. Two strands enter the current cell, one horizontally, the other vertically. In a match cell, the two strands pass through the cell and exit it without crossing (the cell that entered horizontally exits vertically, and vice versa). In a mismatch cell, the strands cross and keep their direction, if and only if the same pair of strands have never crossed before; otherwise, they behave as in a match cell. In AVX-512, this logic can be implemented efficiently by processing the cells in parallel, iterating through the grid in an antidiagonal frontier of independent cells. The frontier is represented by two integer vectors: one storing the indices of the horizontal, the other of the vertical strands. 32-bit integers suffice for all realistic local alignment scenarios. The crossing rules correspond to pairwise sorting of strand indices via vector instruction intrinsics _mm512_mask_min_epu16 / _mm512_mask_max_epu16, using a mask indicating whether individual frontier cells are match or mismatch ones (Table 1). For rational-weighted alignments, the blow-up technique [9] can be used to reduce the problem to the unweighted case.

In future, we plan to extend our implementation to a fast exact local sequence aligner.

**References**

1. P.Krusche and A.Tiskin. Computing alignment plots efficiently. In Parallel Computing: From Multicores and GPU’s to Petascale, vol. 19 of Advances in Parallel Computing series, IOS Press, pp. 158—165, 2010.

2. E.Picot, P.Krusche, A.Tiskin, I.Carré, and S.Ott. Evolutionary analysis of regulatory sequences (EARS) in plants. The Plant Journal, 64(1):165—176, 2010.

3. L.Baxter, A.Jironkin, R.Hickman, J.Moore, C.Barrington, P.Krusche, N.P Dyer, V.Buchanan-Wollaston, A.Tiskin, J.Beynon, K.Denby, and S.Ott. Conserved Noncoding Sequences Highlight Shared Components of Regulatory Networks in Dicotyledonous Plants. The Plant Cell, 24(10):3949–3965, 2012.

4. N.J.Davies, P.Krusche, E.Tauber, and S.Ott. Analysis of 5’ gene regions reveals extraordinary conservation of novel non-coding sequences in a wide range of animals. BMC Evolutionary Biology, 15:227, 2015.

5. "Intel Architecture Instruction Set Extensions Programming Reference". https://software.intel.com/en-us/intel-architecture-instruction-set-extensions-programming-reference

6. A.Tiskin. Semi-local string comparison: Algorithmic techniques and applications. Mathematics in Computer Science, 1, 4, pp. 571—603, 2008.

7. A.Tiskin. Fast distance multiplication of unit-Monge matrices. Algorithmica, 71, 4, pp.859-888, 2015.

8. https://github.com/DimaOrekhov/Seaweed_AVX512

9. Threshold Approximate Matching in Grammar-Compressed Strings. In Proceedings of Prague Stringology Conference, pp. 124—138, 2014.

**Fig. 1 (Abstract O11).**
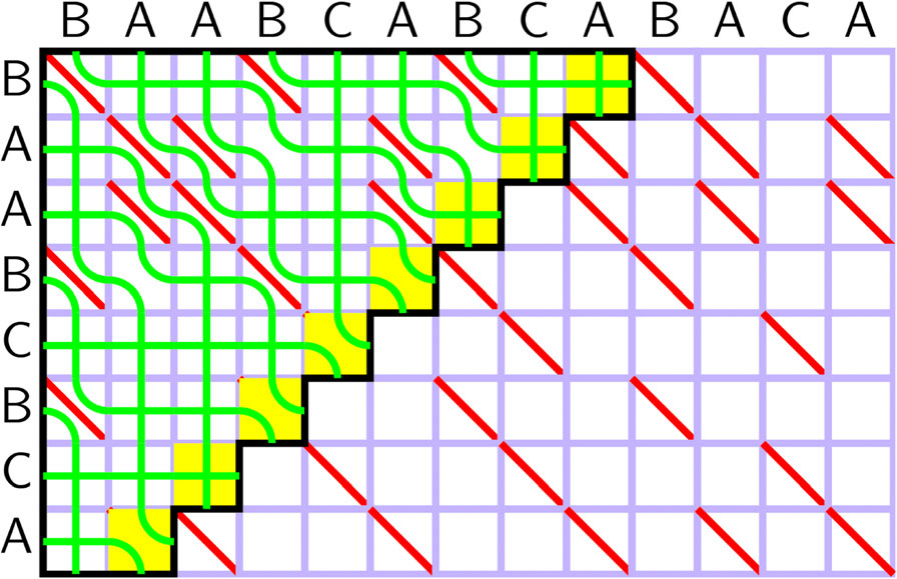
Alignment of pattern “BAABCBCA” vs text “BAABCABCABACA” by braid combing

**Table 1 (Abstract O11).** Section of the inner loop implementing braid combing logic; frontier_h, frontier_v are vectors of 16-bit indices of braid strands entering the frontier horizontally (respectively, vertically)

// obtaining match_mask by comparing pattern_vec vs text_vec: 0 = match, 1 = mismatch match_mask = _mm256_cmpneq_epi8_mask(pattern_vec, text_vec); // combing braid at frontier frontier_h2 = _mm512_mask_min_epu16(frontier_v1, match_mask, frontier_v1, frontier_h1); frontier_v2 = _mm512_mask_max_epu16(frontier_h1, match_mask, frontier_v1, frontier_h1);	

## O12 On the verge of colistin resistance: genetic determinants mediating intermediate colistin resistance in *Klebsiella pneumoniae*

### Sahar Alousi^1^, Tamara Salloum^1^, Balig Panossian^1^, Harout Arabaghian^1^, Rony Khnayzer^1^, George F. Araj^2^, and Sima Tokajian^1^

#### ^1^Department of Natural Sciences, School of Arts and Sciences, Lebanese American University, Byblos, 1401, Lebanon; ^2^Department of Pathology and Laboratory Medicine, Faculty of Medicine, American University of Beirut Medical Center, Beirut, 1107, Lebanon

##### **Correspondence:** Sima Tokajian (stokajian@lau.edu.lb)

**Background**

Colistin is one of the last resort antibiotics used to treat infections by carbapenemase‑producing *Klebsiella pneumoniae* (CPKP). Insertional inactivation of *mgrB,* a gene encoding a negative regulator of the PhoPQ two-component system (TCS), and crrAB (a sensory TCS) have recently gained attention as mediators of colistin resistance.

**Materials and methods**

In this study, broth microdilution colistin susceptibility testing and whole-genome sequencing were used to resolve phenotypic and genotypic resistance profiles in 11 clinical carbapenem‑ and colistin- resistant K. pneumoniae (KP). Whole-genome sequencing (WGS) was performed using short-paired end reads technology on an Illumina Miseq. Core genome single nucleotide polymorphisms (cg-SNP) were called by the Snippy pipeline, and recombination events were highlighted using Gubbins. The pan-genome was generated using Roary. Chromosomally encoded genes were also screened for synonymous and non‑synonymous mutations, in particular, *pmrAB*, *pmrD*, *pmrC*, and *phoPQ*. The genetic environment of *mgrB* was manually validated by Sanger sequencing. Lipid A was extracted using mild acetic acid hydrolysis and profiled using MALDI-TOF MS to examine noteworthy modifications linked to decreased susceptibility to colistin.

**Results**

The lipid A major mass ion was observed at (m/z 1840) in all KP isolates. PCR amplification of *mgrB* revealed insertional inactivation ∆*mgrB* in three of the studied isolates (designated as KP5, KP6, and KP16) showing MICs ≥16 mg/L. IS*Kpn14* was associated with KP5 and KP6, while IS*903* was detected in KP16. Wildtype *mgrB* gene in the remaining 8 isolates might suggest the involvement of other mechanisms underlying their nonsusceptibility to colistin. Recombination analysis highlighted genomic loci involved in both toxin-antitoxin and MFS efflux systems as favored hotspots for recombination. All 11 isolates were negative for the *crrAB* genes. Further biochemical and molecular analysis is in progress to characterize genetic determinants that play key roles in colistin resistance.

**Conclusion**

Along with the escalating prevalence of CRKP and the lack of novel antibiotics, colistin resistance has imposed a worldwide concern. With the power of WGS and lipidomic approaches, genetic alterations in pathways responsible for lipid A modification can be detected with high precision, enabling us to better understand the molecular mechanisms involved in resistance.

## O13 Gene set mining in context relevant Pubmed corpora

### Christophe Van Neste^1,2^, Adil Salhi^1^, Vladimir Bajic^1^

#### ^1^CEMSE, KAUST, Thuwal, 23955-6900, Kingdom of Saudi Arabia; ^2^Department of Biomolecular Medicine, Ghent University, Ghent, 9000, Belgium

##### **Correspondence:** Christophe Van Neste (christophe.vanneste@kaust.edu.sa)

With gene set enrichment analysis, researchers aim to reduce the complexity of their gene-based biological datasets and get more easily interpretable findings as to the functionally relevant differences between experimental conditions. Many methods exist to assess the enrichment of gene sets and make ranked lists out of a collection of gene sets, but they all depend on the coherency of those gene sets in the first place. In general, gene sets are synthesized knowledge from different biological or experimental conditions (tissues, diseases, phenotypes). Only a subset of genes within a gene set might be of relevance for one specific experimental condition or research question. We have developed a literature gene set mining tool, that allows composing a gene set out of genes that are relevant to specific conditions and the research question at hand, by selecting a specific corpus of documents with which to establish the gene set through text mining. After this, the gene set enrichment for that specific set can be analyzed. Furthermore, we include analysis for historic auditing of the gene set. Historic auditing of a gene set allows researchers to see when a gene set became enriched - at a predefined threshold - throughout time in the research niche of their interest, showing the novelty strength of their latest experimental results. We present a specific example: metastasis-related genes for neuroblastoma. Neuroblastoma is a pediatric cancer with a heavy metastasis burden for high-risk patients. However, the type of metastasis is very specific for neuroblastoma and cannot be directly compared to adult metastasized cancers. We show the workflow of mining for the neuroblastoma related gene set of metastasis-relevant genes and analyze its enrichment in neuroblastoma experimental data. As a comparison, we then run a similar analysis on metastatic samples from breast cancer to illustrate the added value of research-specific gene set enrichment analysis. The gene set analysis tool is part of a broader text mining tool “sina” (search indexed nomenclature associations) that we are developing and is available at https://github.com/dicaso/sina.

**Acknowledgements**

C.V.N is funded by Research Foundation - Flanders (FWO) with a postdoctoral fellowship at Ghent University.

## O14 DASE-AG: conditional-specific differential alternative splicing events estimation method for around-gap regions

### Kouki Yonezawa^1^, Ryuhei Minei^2^, Atsushi Ogura^3^

#### ^1^Department of Medical Bioscience, Nagahama Institute of Bio-Science and Technology, Nagahama, Shiga, 526-0829, Japan; ^2^Graduate School of Bioscience, Nagahama Institute of Bio-Science and Technology, Nagahama, Shiga, 526-0829, Japan; ^3^ Department of Animal Bioscience, Nagahama Institute of Bio-Science and Technology, Nagahama, Shiga, 526-0829, Japan

##### **Correspondence:** Kouki Yonezawa (k_yonezawa@nagahama-i-bio.ac.jp), Atsushi Ogura (aogu@whelix.info)

Alternative splicing is a mechanism to generate more than one mRNA isoforms from a single locus, and it increases the genetic diversity during post-transcriptional gene regulation. Furthermore, alternative splicing is often differentially regulated across tissues and during development. It suggests that each splicing isoform may have specific spatial and temporal roles in life system.

We have developed the differential alternative splicing variants estimation method, DASE and DASE2. DASE2 uses FPKMs or TPMs as expression quantities. FPKMs and TPMs are read counts normalized with the lengths of transcripts. However, DASE2 had three problems in finding splicing events. First, splicing events involve gaps in some of the transcripts but DASE2 also considered a series of mismatched nucleotides as splicing events. Second, DASE2 tended to give consecutive gaps at 5’- and 3’-ends higher ranks than those at internal positions. Third, expression quantities of regions around gaps at internal positions are important for detecting splicing events but DASE2 treated expression quantities of whole transcripts. To find alternative splicing (AS) events, for example, intron retention, exon skipping and alternative splice sites, expression quantities of regions including gaps in some of variants and nucleotides in the others are required. We therefore developed DASE-AG for finding series of gaps with their flanking regions with different trends of expressions under the different condition as candidates of AS events. Alternative 5’- and 3’-splice sites found in de novo assembly tend to be more false-positive than skipped exons (SE), retained introns (RI) and mutually exclusive exons (MXE). Therefore, DASE-AG focuses only on series of gaps and their flanking nucleotides, called around-gap regions, and aims to comprehensively detect candidates of SE, RI and MXE.

To assess applicability of our method using RNA-sequence data for estimation of conditional-specific alternative events, we used the RNA-seq dataset of the mouse model of Rett syndrome published by Osenberg et al. in 2018. They focused on intron retentions, exon skippings, and alternative 5’- or 3’-splice sites and reported 114 splicing events with increased inclusion and 65 events with increased exclusion. Among such the events, DASE-AG filtered 7 splicing events up to the 100th rank and DASE2 could not find any of those events. One of the factors is that expression quantities of AG regions tend to be higher than those of the whole sequences of transcripts.

DASE-AG is available at https://github.com/koukiyonezawa/DASE-AG.

## P1 Target selection protocol for DNA-machines development

### Karina P Chalenko^1^, Mikhail S Rotkevich^2^, Dmitry M Kolpashchikov^1,3,4^, Elena I Koshel^1^

#### ^1^Laboratory of Solution Chemistry of Advanced Materials and Technologies, ITMO University, St. Petersburg, Russian Federation; ^2^Theodosius Dobzhansky Center for Genome Bioinformatics, St. Petersburg State University, Saint Petersburg, Russian Federation; ^3^Chemistry Department, University of Central Florida, Orlando, USA; ^4^Burnett School of Biomedical Sciences, University of Central Florida, Orlando, USA

##### **Correspondence:** Karina P Chalenko (karina.p.chalenko@gmail.com)

Deoxyribozymes based DNA-machines are universal approach to cleave mRNA of target gene and can be applied to prokaryotic and eukaryotic organisms. This technique was successfully used against cancer cells and Influenza A Virus [1,2]. Choosing the right target gene is still a fundamental stage for using DNA-machines. In case of eukaryotic organism number of housekeeping genes can reach of thousands, what makes cumbersome subsequent gene analysis by hand.

To achieve goals of our research we developed a Python script utilizing Entrez library, BLAST software [3] and The NCBI SRA Toolkit to access mRNA sequences and estimate the level of gene expression. Firstly, it downloads and creates local indexed databases via ‘sra-toolkit’ [4] and ‘makeblastdb’ applications respectively. Secondly, it queries genes sequences in prepared databases to retrieve summary statistics for their occurrences using ‘blastn’ software. Our software speed up the detection of over-expressed genes, moreover it could deal with both eukaryotic and prokaryotic organisms.

DNA-machine is very sensitive to mismatches in sequences therefore rapid evolution of genes can disrupt the process of mRNA cleaving. MEGA program was used to identify the most conservative genes.

Furthermore, to extend time of work DNA-machines we should choose genes with stable mRNA. This characteristic is determined by the half-life of the mRNA. We exclude genes connected with replication process, which may take a long time, to reduce time *in vivo* experiments.

We verified essentiality of target genes in BioCyc Database Collection demonstrating result of genes knockout. The absence of vulnerable housekeeping gene will lead to cell death.

With the help of the script, we estimated expression level of 3800 housekeeping genes in 2 human transcriptomes. Additionally, we analyzed 206 housekeeping genes in 5 Escherichia coli transcriptomes. On the basis of target selection protocol, we determined the most relevant genes for deoxyribozyme development which are tested *in vitro* (Figure 3). Further, deoxyribozymes based DNA-machines are tested *in vitro* and *in vivo*.

1. Nedorezova D, Fakhardo A, Nemirich D, Bryushkova E, Kolpashchikov D. Towards DNA nanomachines for cancer treatment: Achieving selective and efficient cleavage of folded RNA. Angewandte Chemie. 2019; 131(14): 4702-4706.
2. Solovev Y, Spelkov A, Brushkova E, Kolpashchikov D. Modeling of the DNA-nanodevices for the inactivation of influenza A virus. BiATA.
3. Camacho C, Coulouris G, Avagyan V, Ma N, Papadopoulos J, Bealer K, Madden T L. BLAST+: architecture and applications. BMC bioinformatics. 2009; 10(1): 421.
4. SRA Toolkit Development Team. The SRA Toolkit and SDK from NCBI. http://ncbi.github.io/sra-tools/

**Fig. 1 (Abstract P1).**
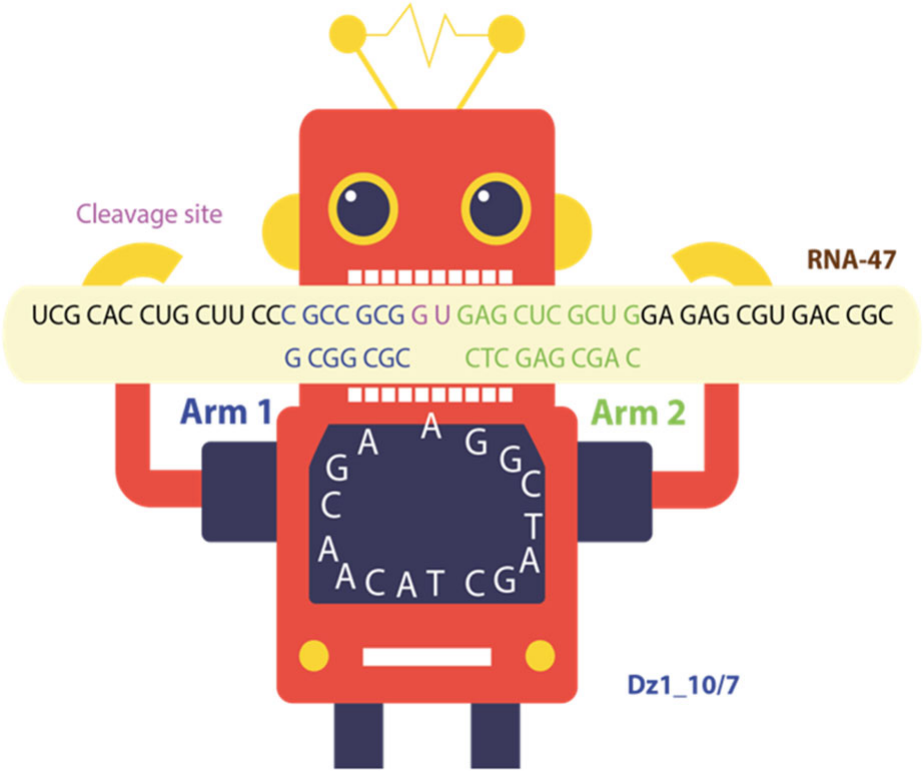
Scheme of deoxyribozyme (Dz) with G/U cleavage site against RNA of streptomycin cassette. Dz has arm 1 and arm 2 with 7 and 10 nucleotides length. RNA contains 47 nucleotides

**Fig. 2 (Abstract P1).**
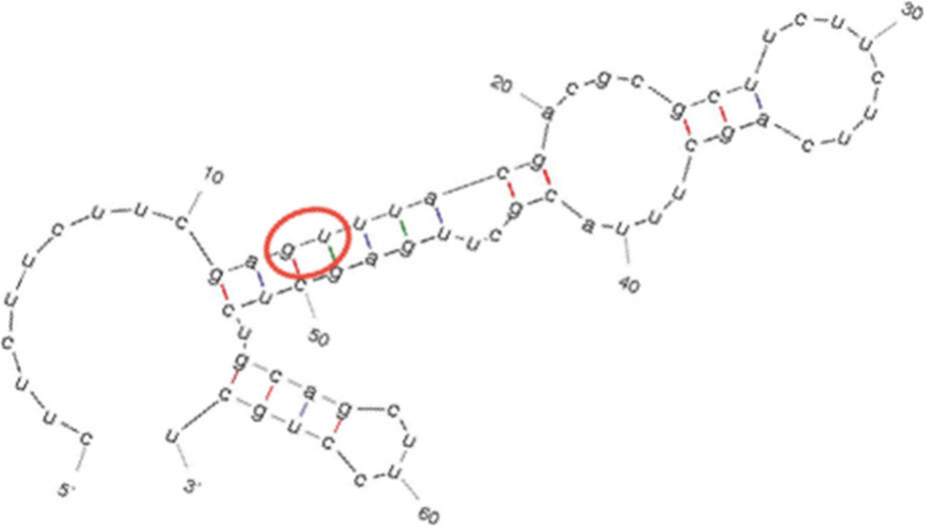
The secondary structure of RNA infB predicted by MFold online tool. G/U was chosen as cleavage site

**Fig. 3 (Abstract P1).**
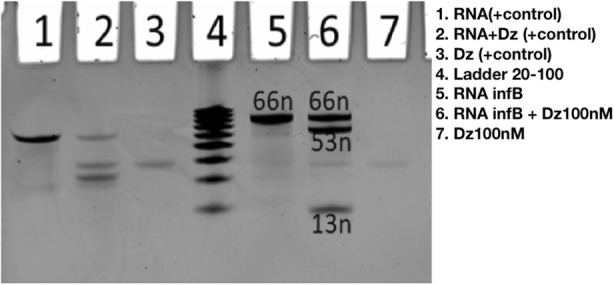
Visualization of RNA infB cleaving results by Dz using ChemDoc after PAGE in 17.5% polyacrylamide gel and Ethidium Bromide staining

## P2 Improved Architecture of Artificial Neural Network for Secondary Structure Analysis

### Semyon Grigorev^1, 2^, Polina Lunina^1, 2^

#### ^1^Saint Petersburg State University, St. Petersburg, 199034, Russia; ^2^JetBrains Research, St. Petersburg, 197374, Russia

##### **Correspondence:** Semyon Grigorev (semyon.grigorev@jetbrains.com)

The idea about using secondary structure while solving different sequences analysis problems is considered in many works [1-4]. One of the classical ways of describing secondary structure is formal grammars.

An approach for biological sequences processing by combination of formal grammars and neural networks is proposed in the work [5]. While classical way is to model secondary structure of the full sequence by using grammar, the proposed approach utilizes it only for primitive secondary structure features description. These features can be extracted by parsing algorithm and processed by neural network. It is shown that this approach is applicable for real-world data processing and several questions are formulated for future research. In this work we provide answers to some of them.

The first question is whether it is possible to use convolutional neural networks for parsing result processing. The result of matrix-based parsing algorithm for some string and fixed nonterminal is an upper-triangular boolean matrix. The first way to represent this matrix is to drop out the bottom left triangle and vectorize the rest of matrix row by row. The vector can be handled by using dense neural network. It requires the equal length of the input sequences, therefore, we propose to cut sequences or add some special symbol till the definite length. The second way is to represent the matrix as an image: the false bits as white pixels and the true bits as black ones, so, we can process sequences with different length and resize images afterwards. To handle these images we use network with a small number of convolutional layers. After linearization we use the same network as for vectors handling.

The second question is whether it is possible to move parsing to network training step, because parsing is the most time-consuming operation of our solution. We solve this problem by using two-staged learning. Firstly, we prepare a network which takes parsed data as an input. After that we extend trained network with a number of input layers that convert original nucleotide sequence into parsing result. This network handles sequences, so, we require parsing only for training the first network.

We use these improvements for tRNA sequences analysis problems: classification into two classes (eukaryotes, prokaryotes) and four classes (archaea, bacteria, plants, fungi). Accuracy for image- and vector based classifiers is about 90% and accuracy for extended networks is about 95% on the test dataset.

**Acknowledgments**

The research was supported by the Russian Science Foundation grant 18-11-00100 and a grant from JetBrains Research.

**References**

1. Rivas E, Eddy SR. The language of RNA: a formal grammar that includes pseudoknots. Bioinformatics. 2000; 16(4): 334-340.

2. Knudsen B, Hein J. RNA secondary structure prediction using stochastic context-free grammars and evolutionary history. Bioinformatics (Oxford, England). 1999; 15(6): 446–454.

3. Yuan C, Lei J, Cole J, Sun Y. Reconstructing 16S rRNA genes in metagenomic data. Bioinformatics. 2015; 12: 135-143.

4. Dowell RD, Eddy SR. Evaluation of several lightweight stochastic context-free grammars for RNA secondary structure prediction. BMC bioinformatics. 2004; 5(1): 71.

5. Grigorev S, Lunina P. The Composition of Dense Neural Networks and Formal Grammars for Secondary Structure Analysis. Proceedings of the 12th International Joint Conference on Biomedical Engineering Systems and Technologies - Volume 3: BIOINFORMATICS. 2019; 234-241.

## P3 Analysis of microbiomes of the ecogenetic series of podzolic soils using an artificial neural network

### Ekaterina Ivanova^1-3^, Elizaveta Pershina^2^, Nadezda Vasilieva^3^, Evgeny Andronov^2,3^, Evgeny Abakumov^1^

#### ^1^Department of Applied Ecology, Biological Faculty, Saint Petersburg state University, Saint Petersburg, 199178, Russia; ^2^ Department of Microbiological Monitoring and Bioremediation of Soils, All-Russia Research Institute for Agricultural Microbiology, Saint Petersburg, 196608, Russia; ^3^ Department of Mathematical Modelling, VV Dokuchaev Soil Science Institute, Moscow, 119017, Russia

##### **Correspondence:** Ekaterina Ivanova (ektrnivanova@gmail.com)

**Background**

The application of molecular-genetic methods is currently one of the necessary steps in the natural microbiomes analysis. The use of these approaches in the study of microbial complexes of soil chronoseries is promising in identifying of taxonomic markers and microbiological drivers of pedogenesis. Otherwise, the search for optimal ways of high-throughput sequencing data processing remains relevant today. The aim of the study was the analysis of the microbial composition of the genetic horizons occurring end developing during the long-term soil evolution in the middle taiga zone.

**Materials and Methods**

Sample set included the podzol soils chronosequences: 1) the initial soil formation (samples of 1-2 years, with no signs of pedogenesis; stages of self-overgrowing (15–20 years with sod-podbur occurrence, 30-35 years old with embryopodzol) and a 2) the ongoing podzol pedogenesis (soils of 70-, 145-, 455, 1590 years). Soil samples were analyzed by classical methods (physic-chemical), DNA isolation was performed using the PowerSoil® DNA Isolation Kit (MO BIO, USA). Amplification of a V4 variable region of the 16S rRNA was carried out with universal primers (F515 and R806) (Bates et al. 2010). Amplicon libraries sequencing was performed by ILLUMINA MiSeq. Sequence data processing was carried out using “Trimmomatic” (Bolger et al. 2014) and “QIIME” (Caporaso et al. 2010) software. Samples clustering and concurrent microbial taxonomic composition analysis was carried out using an artificial neural network that is trained using unsupervised learning - Kohonen's self-organizing map (SOM) (R package ‘kohonen’; Wehrens and Kruisselbrink, 2018).

**Results**

Characteristic patterns of the microbial taxa composition were revealed for the early and late stages of podzolic soils succession. The SOM application allowed to determine the indicative role of microbiome taxonomic structure in marking of solum differentiation during the time of podzol ontogenesis.

**Conclusions**

The neural networks with training is a promising approach to the analysis of soil microbiomes, which in turn helps getting the biologically relevant information (the adaptive and evolutional strategies of microorganisms during soil-formation) and the practically valuable results.

This work was supported by Russian Scientific Foundation (№ 17-16-01030).

References

1. Bolger AM, Lohse M, Usadel B. Trimmomatic: A Flexible Trimmer for Illumina Sequence Data. Bioinformatics. 2014; 30: 2114-2120.

2. Caporaso JG, Kuczynski J, Stombaugh J, et al. QIIME allows analysis of high-throughput community sequencing data. Nature methods. 2010; 7(5): 335-336.

3. Wehrens R, Kruisselbrink J. Journal of Statistical Software. Flexible Self-Organizing Maps in kohonen 3.0. 2018; 87: 7.

## P4 Closing gaps in draft genome assemblies using Oxford Nanopore sequencing and Read-Until technology

### Sergey V. Kazakov^1^, Vladimir I. Ulyantsev^1^, Sergey Nurk^2^

#### ^1^Computer Technologies Laboratory, ITMO University, Saint-Petersburg, Russia; ^2^Center for Algorithmic Biotechnology, SPbSU, Saint-Petersburg, Russia

##### **Correspondence:** Sergey V. Kazakov (svkazakov@corp.ifmo.ru)

For many biological and medical researches it is very important to have a complete genome sequence of organisms included to the study. However, for many of them there are no such sequences, even for organisms thoroughly studied during long time.

While the most reliable sequencing technology both for genome and metagenome projects still remains *Illumina*, the third generation sequencing technologies (such as *Oxford Nanopore* and *PacBio*) become more accessible and wide spread nowadays. They can produce ultra-long reads (hundreds of kbp) that can be used to solve “complicated places” in assembly, originating because of repeats and identical genomes’ parts in different species. Moreover, *Oxford Nanopore* sequencer provides *Read-Until technology* that brings the ability to skip current DNA molecule while reading process is going on! This technology can significantly reduce effective cost of assembly projects.

In current work we proposed several strategies how to use this technology to close gaps in draft genome assembly; in which cases it is reasonable to use it and what benefits one can get using it.

In more detail, we assume that a draft assembly is available for studied organism. Using such fragmented assembly as a reference, it is possible to select only such Nanopore reads, which very likely will connect two or more contigs of assembly.

For experiments we use two datasets with R9 and R9.4 Nanopore reads for *Escherichia coli str. K-12* bacteria. The initial assembly consists of 52 long contigs with 52 gaps between them. Selecting only such reads, we showed that we can close 83% of gaps with 1.9x times more useful reads comparing to the baseline, for the first dataset with R9 reads, and 94% of gaps and 2.0x times more useful reads for second dataset.

It is known that the main problem with such strategies is “short reads” appearing during Nanopore sequencing. Including minimal read length threshold to 5 kbp, enrichment increases up to 2.5x for useful read count with small change in number of covered gaps.

Results for other organisms will also be presented.

## P5 imputeqc: an R package for assession and optimization of genotype imputation parameters

### Gennady V Khvorykh and Andrey V Khrunin

#### Department of Molecular Bases of Human Genetics, Institute of Molecular Genetics of Russian Academy of Sciences, Moscow, Russia

##### **Correspondence:**Gennady V Khvorykh (khvorykh@img.ras.ru)

Genotype imputation increases the power of genome-wide association studies (GWAS). However, not all software for imputation estimates the quality of output. The last release of fastPHASE program (1.4.8) lacks such an option. There is also an uncertainty in choosing the parameters for imputation models. fastPHASE is based on haplotype clusters, where the number of clusters should be set a priori. The choice of the parameter influences the results of imputation and computational time. Besides, this parameter influences the results of the search for genetic signals with hapFLK approach that is based on the same model as fastPHASE.

We present a software toolkit imputeqc to assess the imputation quality of fastPHASE and other softwares. It is based on the masked analysis. The known genotypes are hidden randomly. The data sets are imputed and the genotypes thus obtained are compared to the original ones. The discordance between the genotypes is counted. We demonstrated several applications of this toolkit.

Firstly, it can be applied for benchmarking of imputation software. We applied the tool to the data sets from HapMap and 1000 Genomes Project and compared the quality of imputation made with fastPHASE and BEAGLE softwares. Both programs showed the descordance of about 3%.

Secondly, inputeqc can be applied for choosing the model parameters for imputation with fastPHASE. Two parameters were studied: the number of haplotype clusters and the expectation-maximization cycles. The data set represented merged genotypes of CEU, TSI, CHB, and JPT populations from 1000 Genomes Project. The optimal number of haplotype clusters was estimated to be 20 and the number of expectation-maximization cycles to be 25.

Thirdly, we demonstrated that the tool can be used in conjunction with hapFLK program. The estimated number of haplotype clusters fits well hapFLK model. Applying it to the pool of CEU, TSI, CHB, and JPT population we observed a strong signal of selection at the region of LCT gene. Finally, imputeqc can be applied to estimate the quality of imputation in GWAS by identifying the single nucleotide polymorphism that can be taken for the studies.

The toolkit is implemented as an R package imputeqc and command line scripts. The code is freely available at https://github.com/inzilico/imputeqc under the MIT license.

The reported study was funded by RFBR according to the research project No 19-29-01151.

## P6 CDSnake: Snakemake pipeline for retrieval of annotated OTUs from paired-end reads using CD-HIT utilities

### Yulia Kondratenko, Anton Korobeynikov, Alla Lapidus

#### Saint Petersburg State University, Russia

##### **Correspondence:** Yulia Kondratenko (y.d.kondratenko@spbu.ru)

Sequencing of 16S rRNA is a commonly used method for cost-efficient research of microbial communities. Illumina paired-end reads are often used as sequencing method. Since even short variable regions of 16S provide sufficient information for microbe identification, sequenced fragment is often shorter than sum of lengths of paired reads. Thus reads of pairs can be merged for downstream analysis. In spite of development of several tools for merging of paired-end reads, poor quality at the 3’ ends in the overlapping region prevents the correct assembly of significant portion of read pairs.

Recently CD-HIT-OTU-Miseq was presented as a new approach, avoiding reads merging due to separate clustering of paired reads and discarding of reads voting for non-matching clusters as chimeric. CD-HIT-OTU-Miseq utilities are command line tools written in C++ and Perl. Here we assembled CD-HIT-OTU-Miseq utilities into pipeline using Snakemake workflow. We benchmarked our pipeline with two commonly used pipelines for OTU retrieval, incorporated into popular workflow for microbiome analysis, QIIME2 - DADA2 and deblur. Benchmarking was made on 3 mock datasets, Balanced, HMP, and Extreme, each having highly overlapping paired-end 2 × 250 reads. The Balanced community contained 57 bacteria and archaea at nominally equal frequencies, the HMP community contained 21 bacteria at nominally equal frequencies, and the Extreme community contained 27 bacterial strains at frequencies spanning five orders of magnitude and differing over the sequenced region by as little as 1 nucleotide (nt). CDSnake outputted less OTUs than DADA2 and deblur, since last two tools aim to output sub-OTUs by error processing, and OTU-MiSeq doesn’t process errors and tries to output most correct OTUs using clustering. However, on Balanced and HMP datasets number of OTUs outputted by CDSnake was closer to real number of strains which were used for mock community generation, than those outputted by DADA2 and deblur. On Extreme dataset CDSnake, as expected, performed worse than DADA2 and deblur, since clustering algorithm cannot separate sequencing errors from actual 1-nt differences, present between strains in this community.

CD-HIT-OTU-MiSeq provides one more approach for amplicon analysis capable to outperform popular tools in certain conditions. We developed Snakemake pipeline for OTU-MiSeq utilities, which can be useful for easier automated runs.

This work has been supported by the Russian Science Foundation (grant 19-16-00049)

## P7 Genome heterogeneity affecting binning of complex fungal communities

### Gulnara Tagirdzhanova, Toby Spribille

#### Department of Biological Sciences, University of Alberta, Edmonton, AB, Canada, T6G 2R3

##### **Correspondence:** Gulnara Tagirdzhanova (tagirdzh@ualberta.ca)

The vast majority of fungi are yet to be described and cultured. Since in nature these species mostly occur mixed with other organisms, accessing genomic information from these fungi is a serious challenge. Shotgun sequencing techniques do not offer a reliable way to extract the genome of a target fungus from a mixed dataset, which might include other eukaryotic genomes. Previously, some standard database-independent binning approaches were applied to metagenomes of complex eukaryotic communities. These methods are based on oligonucleotide frequency distribution and rely on the assumption of homogeneity of sequence composition across any given genome. This assumption, however, might not hold true for some fungi. Genomes of these species show strong intragenomic difference in base composition, a phenomenon thought to be caused by repeat-induced point mutation (RIP). RIP is a mechanism used by fungi against transposable elements, silencing multicopy DNA elements by directed mutational processes. Lichens are complex symbiotic communities including multiple species of fungi, algae, and bacteria, and represent a case where two phenomena, unculturable fungi and heterogeneous fungal genomes, overlap. In our study, we aim to assess the extent to which genome heterogeneity might affect metagenomic binning and propose a strategy to improve the binning of complex fungal communities.

## P8 Genome-wide analysis of multidrug-resistant *Shigella* spp*.* isolated from patients in Lebanon

### Yara Salem^1^, Tamara Salloum^1^, Balig Panossian^1^, George F. Araj^2^, Sima Tokajian^1^

#### ^1^Department of Natural Sciences, School of Arts and Sciences, Lebanese American University, Byblos, 1401, Lebanon; ^2^Department of Pathology and Laboratory Medicine, Faculty of Medicine, American University of Beirut Medical Center, Beirut, 1107, Lebanon

##### **Correspondence:** Sima Tokajian (stokajian@lau.edu.lb)

**Background**

*Shigella* spp*.* are Gram-negative rod-shaped bacteria belonging to the family *Enterobacteriaceae* and are a major cause of bacillary dysentery worldwide. In this study, whole-genome sequencing was used for the molecular characterization of ESBL producing *Shigella* spp*.* isolates collected from hospitals in Lebanon.

**Materials and methods**

Polymerase chain reactions (PCRs) were performed to detect β-lactam resistance gene reservoirs and to identify the ones mediating virulence and host adaptation. PCR-based replicon typing (PBRT) was performed to identify patterns of plasmid distribution and multi‑locus sequence typing (MLST), whole‑genome based single nucleotide polymorphism (SNP) analysis, pan-genome analysis and pulse field gel electrophoresis (PFGE) were performed to determine the phylogenic relatedness of the isolates and to trace evolutionary lineages.

**Results**

*S. sonnei* was the dominant serogroup (8/10 *S. sonnei*, 1/10 *S. boydii*, 1/10 *S. flexneri*). A total of 13 genes conferring resistance to aminoglycosides, β-lactams, sulfonamides, trimethoprim, tetracycline and chloramphenicol were identified, while all the isolates were susceptible to ciprofloxacin and norfloxacin. Five types of β-lactamase genes were detected *bla*_CTX-M-15_, *bla*_TEM-1B_, *bla*_OXA-1_ and *bla*_CTX-M-3_, in cephalosporin-resistant isolates. *bla*_OXA-1_ was associated with *S. flexneri*, while *bla*_CTX-M-15,_ *bla*_TEM-1B_, *bla*_OXA-1_ and *bla*_CTX-M-3_ with *S. sonnei*. *bla*_OXA-1_ was linked to class 1 integron integrated on IncFII type plasmid, while *bla*_CTX-M-3_ was detected on an IncI1 plasmid. The genetic environments of *bla*_CTX-M-3_, *bla*_CTX-M-15_ and *bla*_TEM‑1B_ were also determined. All isolates harbored virulence genes and tested positive for the invasion plasmid antigen H (*ipaH*). Serine Protease A (SepA)*,* responsible for critically disrupting the intestinal epithelial barrier, was associated with *S. flexneri*, whereas the invasion associated locus (*ial*) with *S. boydii*. *S. sonnei* had a larger core genome (by approximately 78kb) compared to *S. flexneri* and *S. boydii*, both having a smaller core genome but a wider variety of accessory genes.

**Conclusion**

To the best of our knowledge this is the first detailed molecular characterization of *Shigella* spp. isolates recovered from patients in Lebanon. Our results revealed the association between antimicrobial resistance and increased virulence-related genes, and the emergence of strains with high levels of resistance to third generation cephalosporins. Although there are still some active antimicrobial agents that can be used to treat shigellosis, further emergence of antibacterial resistance by inappropriate use should be carefully followed and prevented.

## P9 Identification of small RNAs derived from commensal microbiota or infections

### Pawel Zayakin^1,2^ (pawel@biomed.lu.lv)

#### ^1^Latvian Biomedical Research and Study Centre, Riga, Latvia; ^2^European Bioinformatics Institute, EMBL-EBI, Hinxton, UK

The intricate mixture of small RNAs of human and non-human origin obtained from a wide range of biofluids is one of the most complex problems to be resolved in RNAseq data analysis. In order to identify accurately those sRNA reads of human origin, the other species sources (bacteria, fungi and viruses) should be separated. For this purpose, we have developed a new algorithm, which allows reducing false positive matching of reads to improper species by two-pass analysis based on the BLAST output on "nr" database using a representable random subset of reads. The second pass will assign the hit to the species, which were most frequently encountered in the first pass, in case of a similar score. At the same time, valuable research information on accompanying species will be also obtained. Only the genomes of the most represented species in successful BLAST hits will be used for the following alignment step. Contrary to full-length mRNA, sRNAs reads usually align in multiple sites of the genome. Our algorithm aligns the reads allowing multiple alignments per read and then reassigns them taking into account the local coverage using ShortStack algorithm.

Results show that, in general, our approach is more suitable for small RNAs analysis than Kraken2/Sourmash/MetaPhlAn2 due to the fact that the K-mers used to generate their databases are longer than most of the small RNA derived reads. Our approach also demonstrates more sensitive results than Kraken2 for highly damaged DNA, as for example, those obtained from archaeological microbiome samples. Nevertheless, the specificity of the method should be improved.

Presented algorithms will be included in the future release of sRNAflow - a software tool for the analysis of small RNAs in biofluids. Besides existing packages for adapter removing, quality control, mapping and counting of reads, differential expression analysis, and miRNA target prediction, this pipeline currently includes the creation of a catalogue of expressed RNA types using human genome annotations and differential expression analysis tools such as DESeq2 for all of the classifiable RNA types. Human genome annotation has been expanded and includes Ensembl database, as well as miRBase, lncipedia, piRBase, piRNAdb, piRNAbank, GtRNAdb and GtRNAdb derived tRFs databases. The prioritization algorithm for building a catalogue of expressed RNA types allows solving the problem that exists when employing different annotations database due to annotations overlap. In addition, our pipeline will include identification of non-templated miRNA isoforms.

